# The Pathology and Treatment of Sexual Impotence

**Published:** 1902-01

**Authors:** 


THE PATHOLOGY AND TREATMENT OF SEXUAL IMPOTENCE. By Victor G. Vecki, M. D. Third edition, revised and enlarged. 12mo., 329 pages. Philadelphia and London: W. B. Saunders & Company. 1901. Cloth, $2.00 net.

Whatever else may be said of it, this little volume is at least readable. The author's particularly attractive style would make it interesting even if it contained less of real value. Many will not agree with him that sexual impotence is often worse than death, or even that a lack of virility totally unfits a man for the ordinary duties of a business or professional life. However, no one will be likely to deny that impotence, both partial and complete, is becoming a comparatively frequent disease in the centers of civilization, and that its effects upon society are pernicious and far-reaching.

The book has good reason for being. It deserves a careful reading by every thinking member of the profession. R.

